# Progress in Surgery

**Published:** 1895-02-02

**Authors:** 


### Progress in Surgery.

### GENERAL SURGERY.

**Antiseptics.**—In his presidential address before the Bristol branch of the British Medical Association Nelson C. Dobson[1] said: "If I were asked to name what I consider the greatest advance of modern surgery, I should unquestionably say, taken in all its far-reaching influences—in enormously lessening the rate of mortality, in reducing the period of convalescence, in robbing operations of many of their terrors to the surgeon, and of much of their pain to the patient—the antiseptic treatment of wounds." These words have great weight, coming as they do from a surgeon who has been for more than twenty-five years actively engaged in the practice of surgery. Pus, surgical pyrexia, and pyæmia have almost entirely disappeared, and are replaced by almost certain primary healing of wounds. However much the methods we may adopt for carrying out our ends may vary (he adds prophetically), the antiseptic principle will remain with us so long as surgery has any existence. Drainage, proper suturing of the wound, absorbable ligatures, the environment of the patient, etc., no doubt have largely contributed to this great advance, but the destruction and exclusion of micro-organisms plays the most important part. Langley Browne[2] gives a short but lucid account of the development of the germ theory. Starting from the time of Linnæus, who published an article in 1730 attributing small-pox, measles, plague, syphilis, dysentery, and whooping cough to the agency of minute animals, he passes in review Ehrenberg's discoveries in 1828, Schwann's views in 1837, Sir Henry Holland's "Hypothesis of Animalcule Life as a Cause of Disease" in 1839, Libermeister's introduction to "Infectious Diseases" in Ziemssen's "Cyclopædia of Medicine," 1875, Henle's theory of a contagium vivum in 1853, down to recent times. The labours of Pasteur, who made the earliest discoveries in bacteriology, of Koch, Klein, Klebs, Nicolaier, Lemaire, and Metschnikoff are all mentioned. Lastly, Lister, with a combination of experimental resource, patience, and brilliancy almost unparalleled in the history of surgical science, step by step built up the modern theory and practice of antiseptic surgery. Surgery and its subjects owe to Lister a debt which can never be repaid. Leedham-Green[3] discusses the two points of the highest importance, which should always be kept in view in the treatment of wounds—(*a*) the preservation of the vitality of the tissues of the wound; (*b*) the exclusion from the wound, as far as practicable, of all bacterial life. As to the former, he believes that washing the wound with weak or strong antiseptic solutions, even with simple distilled water, is injurious, as it causes an actual necrosis of the superficial cells. If a recent wound is inoculated with disease germs, all subsequent irrigation (even after a short time) with antiseptics appears according to the recent researches of Schimmelbusch[4] to be powerless to ward off a fatal result. If the drainage is to be dispensed with the following points are specially noted by Leedham-Green: (1) The most rigid aseptic precautions. (2) The exact arrest of all hæmorrhage. (3) The avoidance of all irrigation. (4) Gentle but firm pressure by means of careful dressings, so as to bring the cut surfaces into contact with one another. There are two principal sources of infection to be guarded against, viz., (1) The surrounding atmosphere, and (2) Contact with infected objects. The re-examination by German investigators of Schimmelbusch's experiments on the disinfection of fresh wounds[5] confirms them fully. According to this, the uselessness of disinfection after infection with "septic disease," so-called by Schimmelbusch, cannot be doubted.

Although a faultless asepsis enables us to do away with irrigation and drainage, yet under a number of circumstances antiseptics must still be extensively employed. Frere[6] draws attention to one of the most powerful germicides known at present, viz., biniodide of mercury dissolved in iodide of sodium, so ably advocated by Illingworth, Loekwood, and others. Until quite recently it has been more or less disregarded. Frere gives an easy mode of preparation of the solution by adding a solution of iodide of potassium and sodium to the liq. hydrarg perchlor B.P. until the precipitate which forms is dissolved. This saves an immense amount of time, trouble, expense, and anxiety to the general practitioner. Having washed the wound with the solution (1 in 1,000) it is sufficient to keep lint applied to the part constantly moist with the same lotion of the strength of 1 in 2,000. Pendleton[7] gives a useful list of points to be remembered in the practice of antiseptic surgery.

For the surgical care of the nails, O. H. Allis[8] recommends a wedge-shaped piece of rubber eraser instead of the nail brush, A medium length of nail, he says, is an exceedingly valuable helper at times in surgical operations. If too long they are in the way, and if too short a privation in prehension, &c. Professor Terrier[9] is convinced that it is useless to attempt securing asepsis in the field. While thoroughly in favour of asepsis in civil practice, he is equally opposed to anything but antisepsis in military surgery. The best method of disinfecting walls of apartments, according to Laveran,[10] consists in washing them first with soap and water, and then with a 5 per cent. carbolic acid, or a 2 per 1,000 corrosive sublimate solution. This is preferable to applying antiseptic solutions in spray. Sulphur is considered by Sage[11] to be an agent of doubtful utility in disinfection. Its action does not penetrate to the degree formerly supposed, but it acts upon the surface only. In a recent number of THE HOSPITAL A. G. Miller[12] gives his experience of the uses of carbolic acid beyond the cleansing of the skin of patients and the hands of surgeons for operations. Its action is threefold — astringent, anæsthetic, and antiseptic—and is useful in whitlow, septic puncture, simple traumatic synovitis or acute bursitis, phlebitis, inflamed external piles, sprains, and bruises. Internally he has used it for typhoid fever and erysipelas. The effect of iodoform on the tissues has recently been exhaustively studied by Stubenrauch.[13] The author concludes that iodoform is not an antiseptic in the true sense of the term, as is corrosive sublimate, inasmuch as it does not destroy bacteria by short contact. It has also no specific anti-tuberculous (anti-bacterial) effect. The favourable results in cases of chronic tuberculous disease of glandular organs, and other pathological new formations is due to its decomposition. On tuberculous tissue iodoform acts probably by hastening the decay of the epithelioid cell collections which are destined to degeneration, while the more resistant of the epithelial elements are prepared for a change into healthy tissue. Acetanilide (antifebrin) has been used by Woods[14] as a substitute for iodoform. He has employed it in twenty cases of operative wound and laceration and in internal hæmorrhoids. As an injection in gonorrhœa he recommends ℞ acetanilide ʒj, alcohol ʒss, aqua ad. f. ℥ viii. Blagoveshtchensky's experiments[15] fully support Klein's high opinion of the antiseptic properties of Izal. Eberth's typhoid bacillus is killed by a 3 or 5 per cent. mixture in three minutes, while pyogenic microbes lose their vitality after a few minutes' contact with a 2 per cent. emulsion. Even very strong emulsions of the substance are absolutely harmless. Referring to this paper, Diakonoff,[16] of Moscow, points out that commercial specimens of izal are found to vary in their chemical composition, while another disadvantage consists in its being non-transparent. Stabel[17] believes that diaphtherin is considerably superior to lysol and carbolic acid as a germicide. It is especially adapted on account of its non-toxic properties to washing out hollow cavities where very weak antiseptics have to be employed. It is preferable in one or two per cent. solutions to other antiseptics, where moist applications are required for a long time, as burns, ulcers, &c. Steel instruments cannot be put into it. Two new and cheap dressings for wounds have lately been introduced, viz., peat-wool and straw-coal. The former has been used by Lucas-Championnière,[18] and other French, German, and Russian surgeons, on account of its reputed antiseptic qualities, combined with its great power of absorption and permeability. The French War department has definitely adopted it to replace gauzes and wools. The latter substance, obtained by burning rice straw, has been resorted to by Dr. Kikuzi[19] at Tokio with great advantage, being perfectly aseptic, very soft, porous, and elastic. It cannot be directly applied to the wound, and must therefore be enclosed in disinfected linen bags. In this connection we may allude to an interesting case recorded by Irvine,[20] illustrating the value of the aseptic blood clot as a surgical dressing. The injury was a compound comminuted fracture of both bones of the leg in a boy, with rupture of the anterior tibial artery. The clot which formed about the wound resulted from hæmorrhage after setting of the fracture, and was allowed to remain. On the ninth day the last shred of the clot came away without any signs of putrefaction, and a most useful limb ultimately resulted.

[1] Pamphlet, Presidential Address, British Branch Brit. Med. Assn. June 27th, 1894, p. 5. [2] Birmingham Medical Review, Aug., 1894, p. 65. [3] Ibid., July, 1894, p. 14. [4] Brit. Med. J., Aug. 18th, 1894, and Deut. Med. Woch, July 12th, 1894. [5] Central f. Chir., 1894, No. 30, and Amer. Med. Surg. Bulletin, Sept. 15th, 1894, p. 1,130. [6] The Hospital, July 7th, 1894, p. 297. [7] Med. Record, N. Y., Sept. 15th, 1894, p. 333. [8] N. Y. Med. Jour., July 21st, 1894, p. 81. [9] Medical Week, June 29th, 1894, p. 308. [10] Ibid., July 27th, 1894, p. 353. [11] Therapeutic Gazette, Sept. 15th, 1894, p. 633. [12] The Hospital, June 23rd, 1894, p. 252. [13] Amer. J. of Med. Sciences, Aug., 1894, p. 233. [14] Therapeutic Gazette, Sept. 15th, 1894. [15] Ibid., Sept. 15th, 1894. [16] Vratch, No. 5, 1894. [17] Canadian Practitioner, April, 1894, p. 281, and Med. Chronicle, Aug., 1894. [18] Therapeutic Gazette, Sept. 15th, 1894. [19] Medical Week, Aug. 3rd, 1894. [20] Edinburgh Med. Jour., Aug., 1894, p. 114.

